# S59. CHILDHOOD TRAUMA IS ASSOCIATED WITH SOCIAL COGNITION AND SCHIZOTYPAL PERSONALITY TRAITS IN PSYCHOTIC AND HEALTHY POPULATIONS

**DOI:** 10.1093/schbul/sby018.846

**Published:** 2018-04-01

**Authors:** Yann Quide, Sarah Cohen-Woods, Nicole O’Reilly, Vaughan Carr, Bernet Elzinga, Melissa Green

**Affiliations:** 1 University of New South Wales; 2 Flinders University; 3 Leiden University; 4 University of New South Wales, Prince of Wales Hospital

## Abstract

**Background:**

Childhood trauma is a transdiagnostic risk factor for adult psychiatric disorders, including schizophrenia and bipolar-I disorder. Recent meta-analytic and epidemiological studies suggest a 3-fold increase in risk for psychotic symptoms in adulthood, following childhood trauma exposure. However, associations between trauma exposure and schizotypal personality traits, as well as cognitive and social cognitive abilities, have been less well studied in clinical populations spanning the psychotic-mood spectrum.

**Methods:**

Participants were 79 schizophrenia cases, 84 bipolar disorder cases, and 75 healthy control participants who completed the Childhood Trauma Questionnaire (CTQ), the Schizotypal Personality Questionnaire (SPQ), and a standard battery of cognitive tests (to measure executive functions, working memory, attention, immediate and delayed memory), as well as social cognitive tests of facial emotion processing (the Ekman 60 faces task) and Theory-of-Mind (The Awareness of Social Inference Test; TASIT). The CTQ measures childhood trauma exposure on 5 domains (physical abuse, emotional abuse, sexual abuse, physical neglect, emotional neglect); clinically significant levels of childhood trauma exposure on at least one domain (according to specified thresholds for each domain) were evident in 54 schizophrenia cases, 55 bipolar disorder cases, and 26 healthy individuals. Trauma-exposed and non-exposed groups were compared on schizotypal personality features (referred to as ‘schizotypy’), cognitive and social cognitive abilities.

**Results:**

In both the clinical groups and healthy controls, trauma-exposed participants reported higher levels of schizotypy, especially suspiciousness, relative to non-exposed individuals; this was revealed in the context of higher overall schizotypy levels in both schizophrenia and bipolar disorder, relative to healthy controls. Similarly, while the schizophrenia group showed lower social cognitive and cognitive performances relative to both the bipolar disorder and healthy control groups, trauma-exposed individuals showed deficits in social cognitive, but not general cognitive abilities, regardless of case versus control status.

**Discussion:**

These findings suggest that childhood trauma exposure has long-term effects on schizotypy, especially suspiciousness, and complex social cognitive abilities in both healthy and psychotic populations. However, there was no interaction of clinical group with trauma exposure in relation to schizotypal personality dimensions, and the influence of early life trauma on cognitive functions was not distinguishable from the effects of psychotic illness in adulthood. It is possible that traumagenic processes contribute to paranoid ideation and social cognitive disturbances that contribute to psychosis-proneness in the general population, consistent with historical models of schizotypy as latent liability for schizophrenia and related psychotic disorders.

